# Refining experimental dental implant testing in the Göttingen Minipig using 3D computed tomography—A morphometric study of the mandibular canal

**DOI:** 10.1371/journal.pone.0184889

**Published:** 2017-09-14

**Authors:** Giuliano M. Corte, Johanna Plendl, Hana Hünigen, Kenneth C. Richardson, Ole Gemeinhardt, Stefan M. Niehues

**Affiliations:** 1 Institute of Veterinary Anatomy, Department of Veterinary Medicine, Freie Universität Berlin, Berlin, Germany; 2 College of Veterinary Medicine, School of Veterinary and Life Sciences, Murdoch University, Murdoch, Western Australia; 3 Department of Radiology, Charité – Universitätsmedizin Berlin, Berlin, Germany; Universidad de Zaragoza, SPAIN

## Abstract

This study reports morphometric and age-related data of the mandibular canal and the alveolar ridge of the Göttingen Minipig to avoid complications during *in vivo* testing of endosseus dental implants and to compare these data with the human anatomy. Using 3D computed tomography, six parameters of the mandibular canal as well as the alveolar bone height and the alveolar ridge width were measured in Göttingen Minipigs aged 12, 17 and 21 months. Our null hypothesis assumes that the age and the body mass have an influence on the parameters measured. The study found that the volume, length and depth of the mandibular canal all increase with age. The width of the canal does not change significantly with age. The body mass does not have an influence on any of the measured parameters. The increase in canal volume appears to be due to loss of deep spongy bone in the posterior premolar and molar regions. This reduces the available space for dental implantations, negatively affecting implant stability and potentially the integrity of the inferior alveolar neurovascular bundle. Dynamic anatomical changes occur until 21 months. On ethical grounds, using minipigs younger than 21 months in experimental implant dentistry is inadvisable. Paradoxically the measurements of the 12 months old pigs indicate a closer alignment of their mandibular anatomy to that of humans suggesting that they may be better models for implant studies. Given the variability in mandibular canal dimensions in similar age cohorts, the use of imaging techniques is essential for the selection of individual minipigs for dental prosthetic interventions and thus higher success rates.

## Introduction

Because of their similar anatomy and physiology to that of humans the Göttingen Minipig is often used as a large animal model [1] in areas of research such as toxicology [2], neuroscience [3] diabetes [4] and obesity studies [5, 6]. Their small size, rapid growth and early sexual maturity allow easier handling and more economic housing, features that make them preferable to normal-sized pigs or other large animal species for long-term studies [7, 8]. Miniature pigs are used frequently as an animal model in dental research [9] because of their heterodont dentition with incisors and molars only slightly larger than in humans. Their being diphyodont and having similar eruption patterns validate their suitability as an animal model for dentistry [10–12].

Over recent decades, public concern about animal welfare has evolved gradually resulting in 2010 with the European Union issuing Directive 2010/63/EU for the implementation of the 3R concept i.e., replace, reduce, refine [13] in research [14]. The aim of the refine-principle is to modify animal testing to minimize distress, pain and suffering using improved experimental techniques [15].

This is particularly so when testing new dental implants and biomaterials in animal models. Here it is important to have detailed knowledge of the animals’ facial anatomy. The knowledge of interspecific differences and of intraspecific anatomical variability in breeding lines, improves the outcomes of these surgical interventions and lessens the possibility of failure or of potentially misleading and meaningless results with limited transferability [16].

The mandibular canal (Canalis alveolaris inferior) originates at the foramen mandibulae and runs within the substance of the mandibular body to terminate immediately rostral to the first premolar tooth [17]. The canal conveys the inferior alveolar neurovascular bundle, which consist of the inferior alveolar artery and vein and the inferior alveolar nerve [18]. In recent times, dental research has often involved *in vivo* testing of endosseus dental implants in 12–24 months old Göttingen Minipigs [19–21]. In some cases, these interventions have failed entirely or have had less than satisfactory results. Often this has been due to implant instability. An additional procedural problem has been the penetration of the mandibular canal during experimental procedures resulting in injury to the inferior alveolar neurovascular bundle causing bleeding, swelling, neurosensory alterations like paraesthesia, hyperaesthesia or dysaesthesia and pain [22–25].

Although detailed morphometric data is important to the success of oral implant surgeries, only a few studies of miniature pigs’ mandibular morphometry exist. Consequently, this study was designed to provide detailed morphometric and age-related *in vivo* data of the mandibular canal and the alveolar process of the Göttingen Minipig using 3D computed tomography. We measured the volume, length, depth, width and inferior bone thickness of the mandibular canal as well as the alveolar bone height and the alveolar ridge width. We also focused on the configuration and course of the inferior alveolar neurovascular bundle within the mandibular canal.

Our null hypothesis is, that there are no significant differences between the left and right hemimandibles (mandibular halves) and that the age and body mass have an influence on the parameters measured.

## Material and methods

The CT data sets were created in 2007 and 2008 in the course of another research study, that was approved by the Regional Office for Health and Social Affairs Berlin (permit IC113-G 0281/12) and was conducted at the medical faculty (certified by ISO 9001) of the Charité Campus Virchow-Klinikum, Berlin. [26]. The reuse of the data is in accordance with the 3Rs, but precluded an optimal study design, however it was an opportunity to further our knowledge base on the little known mandibular morphology of the Göttingen minipig.

### Animal groups and husbandry

A total of 18 healthy female Göttingen Minipigs consisting of six animals examined at the age of 12 months (12m; n = 6; 357±31d) and 12 animals examined at an age of 17 months (17m; n = 12; 511±24d) and again at the age of 21 months (21m; n = 11; 620±37d). The animals’ weight ranged from 23 to 44 kg. In the 21-month group (adult animals), one animal was excluded due to the loss of some of its data.

The minipigs were obtained from Ellegaard, Göttingen Minipigs^®^ (Dalmose, Denmark) where they had been habituated to routine handling by humans to lessen the effects of humans as stressors in their daily life.

Subsequently, at the research facility in Berlin, animals were held according to the Guidelines of the European Societies of Laboratory Animal Science. The pigs were grouped into pens of six animals, with a light/dark rhythm of 12/12 hours, a relative humidity of 55 ± 10% and temperatures between 15 and 24°C. The animals were fed a restricted diet designed for minipigs (Ssnif Spezialdiäten GmbH, Soest, Germany) to prevent obesity [27]. Their body mass was measured weekly.

### Computed tomography

#### Anaesthesia and drug administration

Prior to tomography, animals were fasted for 24 hours with water *ad libitum*. Then the animals were premedicated by intramuscular injection of 0.5 mg atropine (Atropinum sulfuricum, 1 mg/ml, Eifelfango, Bad Neuenahr-Ahrweiler, Germany). Anaesthesia was induced by intramuscular injection of ketamine (27 mg/kg, Ursotamin^®^, 100 mg/ml, Serumwerk Bernburg, Germany), xylazine (3.5 mg/kg, Rompun^®^ TS, 20 mg/ml, Bayer Vital GmbH, Leverkusen, Germany) and 3 ml azaperone (Stresnil^®^, 40 mg/ml, Janssen Animal Health, Neuss, Germany). An electrolyte solution was infused intravenously throughout the entire procedure (Ionosteril^®^, Fresenius, Bad Homburg v.d.H., Germany) [28]. At the end of the experiment all animals were euthanised in deep anaesthesia by intravenous injection of 15 ml T 61 (Intervet Deutschland GmbH, Unterschleißheim, Germany) for separate studies of the vascular distribution of the whole body [26] and histologic experiments.

#### Equipment and software

Data acquisition was performed on a 64-slice scanner (Lightspeed 64^®^; GE Medical Systems, Milwaukee, USA). For contrast enhancement, 80 ml of a nonionic iodinated contrast medium (XenetiX^®^ 350, Guerbet GmbH, Sulzbach, Germany 350 mg iodine /ml) with automatic intravenous injection of 4 ml/sec was injected through the lateral ear vein of every pig. The examination timing was multiphasic, bolus arterial triggered and venous (with an 80 sec delay). Scan parameters were standardized (voltage of 120 kV, maximal 500 mA with automatic mA-optimization at a noise index of 15, mean 490 mA; collimated slice thickness of 64×0.625 mm; total detector width of 55 mm; rotation speed of 0.4 sec; table feed per rotation: 55 mm) [26]. The scan field of view (SFOV) was 50 cm and the display field of view was 39.7 cm. The physical detector width covers 40 mm in z-axis and the used pitch factor was 1.375. The positioning and the following computed tomographic examination required only a few minutes per animal. The 12m pigs were imaged twice at an interval of 27 days, whilst the 17m and 21m pigs were imaged five times over 111 days. Then the data was transferred to an independent workstation using the software Vitrea Advanced^®^ 6.6 (Vital Images Inc., Minnetonka, MN, USA). Volumetric assessment was reconstructed without overlap of images with a slice thickness of 1.25 mm.

### Parameters measured

To identify landmarks and to ensure high reproducibility, multiplanar (sagittal, coronal, axial) views, reconstructed from the original axial slices were used [29]. In addition, bone reconstruction kernels were used (Bone plus, GE Medical Systems, Milwaukee, USA).

All parameters were measured on both left and right hemimandibles. The volumes are given in millilitres (ml), all other parameters in millimeters (mm).

**Fig 1:**
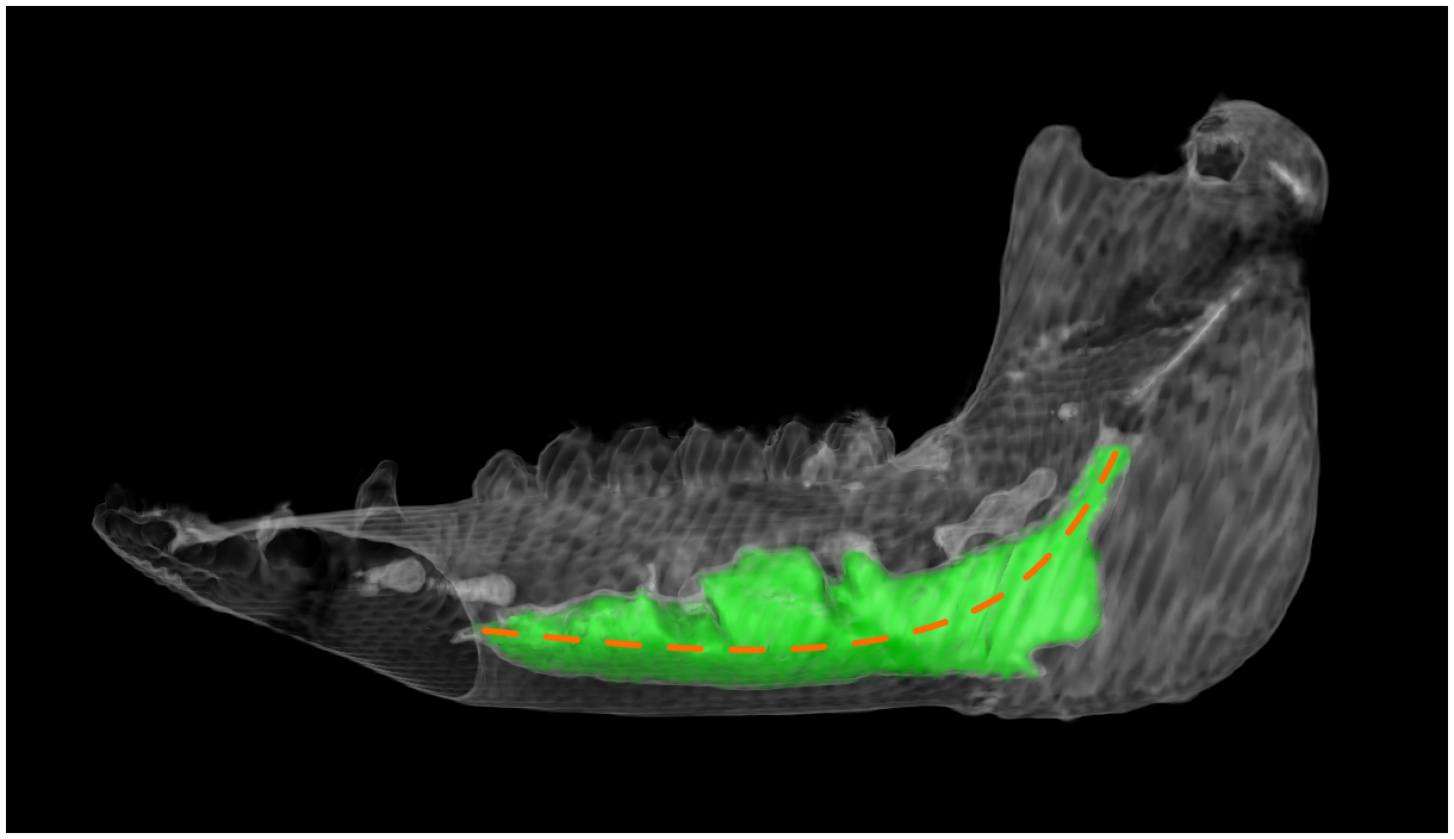
The mandible of a 17m old pig showing the volume of the mandibular canal. The volume of mandibular canal (VCM) appears in green and the length of mandibular canal (LCM) as the dashed red line.

To measure the mandibular canal volume (VCM) the “vessel grow” function of Vitrea Advanced^®^ was utilized to segment the mandibular canal and to calculate its’ volume. The short canals branching off the main canal and forming the numerous mental foramina in pigs were excluded from the volume calculations (Fig 1).

The length of the mandibular canal (LCM) in each hemimandible was measured using images of the axial plane where the software connected the midpoints at each segmentation level along the entire length of the canal, forming a continuum (Fig 1).

**Fig 2:**
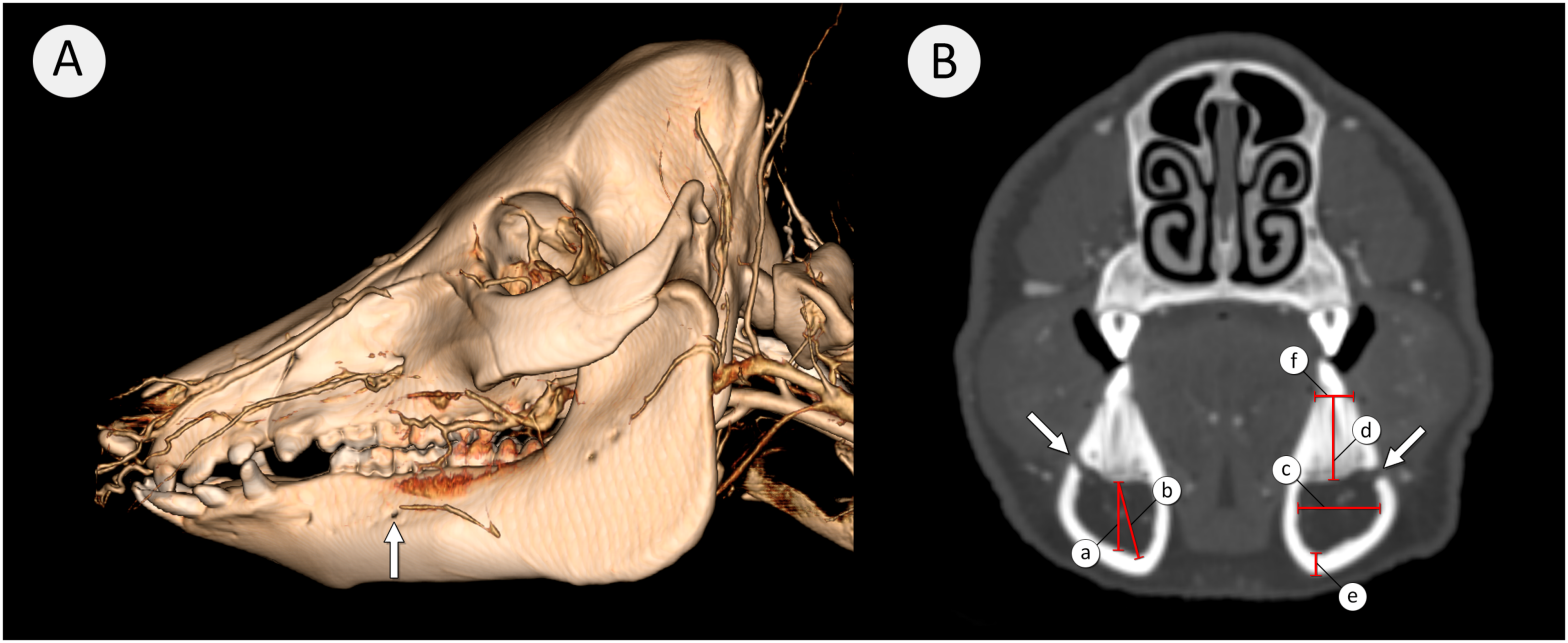
3D rendering of the head of a minipig (A) and a transverse section view at the level of the posterior mental foramen (B). (A) Arrowed is the prominent posterior mental foramen. Image (B) shows the measured parameters with (a) Maximal vertical depth, (b) Maximal oblique depth, (c) Maximal width of mandibular canal, (d) Alveolar bone height, (e) Inferior bone thickness and (f) Alveolar ridge width. The white arrows indicate the posterior mental foramen.

All subsequent parameters (Fig 2B) were measured at the level of the most posteriorly located mental foramen (Fig 2A). This consistent anatomical landmark facilitated the comparison between the age groups.

The maximal vertical depth of the mandibular canal (MVD) was determined by drawing a vertical line from the midpoint of the superior aspect of the mandibular canal opposite to the inner surface of the canal.

The maximal oblique depth of the mandibular canal (MOD) was determined by drawing an oblique from the same superior midpoint to the most distal inferior point. If the MVD was less than MOD, the shape approaches an oval. If the MVD equaled the MOD, the canal was assumed to be circular.

The width of the mandibular canal (WCM) was defined by the largest horizontal measurement between the buccal and lingual aspects of the canal.

The alveolar bone height (ABH) was determined by drawing a vertical line from the midpoint of the superior aspect of the canal to a horizontal line connecting the buccal and lingual alveolar crests.

The inferior bone thickness (IBT) was measured by drawing a line from the most distal inferior point of the canal across the broadest bony dimension to the most inferior point of the mandibular body.

The alveolar ridge width (ARW) was measured by the length of a line connecting the buccal and lingual alveolar crests.

### Dissection

To confirm that the inferior alveolar nerve runs in close proximity with the inferior alveolar vessels and to determine its’ position within the mandibular canal, two hemimandibles of an adult Göttingen minipig were dissected. The mandible was a donation to the Institute of Veterinary Anatomy, Freie Universität Berlin. The mandible was transected by a saw cut through the mandibular symphysis into its’ hemimandibles. All the external soft tissues were dissected from the hemimandibles. Subsequently one hemimandible was sawn transversally at 1 cm intervals along its entire length. In the other hemimandible, the mandibular canal was exposed from the medial aspect using a micro milling tool (Micromot^®^, Proxxon GmbH, Föhren, Germany) from the mandibular foramen to the first molar tooth. Any fatty tissue within the canal was removed to clearly visualise the inferior alveolar neurovascular bundle.

### Statistics

For statistical analysis we used IBM SPSS Statistics 23 (IBM Deutschland GmbH, Kassel, Germany). Every parameter was checked for normality. If normal distribution could be assumed, we used the student’s *t*-Test and for non-normal data the Mann-Whitney-U, Wilcoxon and Kruskall-Wallis Test. When comparing 12m animals with animals of 17m and 21m, the Independent T-test was used. This was because the animals in the 12m group differ to those of 17m and 21m group. However, animals in 17m and 21m group were the same individuals and therefore paired samples. For this statistical comparison the Paired-student’s *t*-Test was used. The correlations between parameters were analyzed with a bivariate Pearson-Test and Spearman-Rho-Test, depending on the distribution of the data. The values are given as mean values with the associated standard deviation. A *p* value of less than 0.05 was considered significant. A correlation coefficient (r) between 0.45 to 0.59 was considered to be a moderate correlation, whereas a correlation coefficient between 0.60 to 0.79 was perceived to be a strong and from 0.80 to 1.0 to be a very strong correlation.

All measurements were conducted by the same trained examiner and under the supervision of an experienced radiologist. To estimate the observers’ reproducibility of the measured values, several blind tests were conducted. A mean percentage standard deviation of 2.2% proved that the measurements were executed precisely and were therefore reliable.

## Results

Table 1 shows the mean values and standard deviations of all measured parameters. The correlation coefficient between left and right side, correlation with age and with body weight are in Table 2.

**Table 1:** Mean values and standard deviations of all measured parameters. Because data from all measure of the left and right hemimandibles were statistically similar, data from the left and right hemimandibles were pooled for this table.

Age group (months)		Volume of the mandibular canal [ml]	Length of the mandibular canal [mm]	Maximal vertical depth of the mandibular canal [mm]	Maximal oblique depth of the mandibular canal [mm]
12 months (n = 6)	Mean	**3.71**	**94.29**	**7.60**	**7.98**
	Std. Deviation	0.79	4.82	2.00	1.65
17 months (n = 12)	Mean	**6.86**	**104.13**	**9.78**	**11.37**
	Std. Deviation	2.26	3.65	1.63	2.18
21 months (n = 11)	Mean	**8.27**	**108.58**	**11.74**	**12.44**
	Std. Deviation	2.58	3.93	1.46	1.91
Age group (months)		Width of the mandibular canal [mm]	Alveolar bone height [mm]	Inferior bone thickness [mm]	Alveolar ridge width [mm]
12 months (n = 6)	Mean	**10.32**	**17.74**	**4.65**	**7.20**
	Std. Deviation	0.60	2.73	0.88	1.10
17 months (n = 12)	Mean	**11.21**	**13.99**	**3.65**	**8.26**
	Std. Deviation	1.17	1.20	1.20	0,86
21 months (n = 11)	Mean	**11.59**	**14.33**	**3.95**	**7.85**
	Std. Deviation	1.65	1.16	1.14	0.82

**Table 2:** Overview of the correlation between left and right hemimandibles, correlation with age and with body weight. A correlation coefficient (r) between 0.45 to 0.59 was considered to be a moderate correlation, whereas a correlation coefficient between 0.60 to 0.79 was considered to be a strong and from 0.80 to 1.0 to be a very strong correlation. The significance levels are reported as *p<0.05; **p<0.01; ***p<0.001; ^ns^ = p>0.05.

Parameter	Correlation between left and right hemimandible	Correlation with age (days), left/right hemimandible	Correlation with body mass (kg), left/right hemimandible
VCM	r = 0.994***	r = 0.616**/0.579**	r = 0.174^ns^/0.145^ns^
LCM	r = 0.883***	r = 0.783**/0.701**	r = 0.309^ns^/0.293^ns^
MVD	r = 0.892***	r = 0.613**/0.755**	r = 0.302^ns^/0.394^ns^
MOD	r = 0.913***	r = 0.618**/0.689**	r = 0.112^ns^/0.249^ns^
WCM	r = 0.511**	r = 0.282^ns^/0.170^ns^	r = 0.022^ns^/-0.126^ns^
ABH	r = 0.866***	r = -0.536**/-0.451*	r = -0.075^ns^/-0.031^ns^
IBT	r = 0.908***	r = -0.128^ns^/-0.063^ns^	r = -0.108^ns^/-0.010^ns^
ARW	r = 0.737***	r = 0.058^ns^/0.072^ns^	r = -0.010^ns^/-0.184^ns^

### Volume of the mandibular canal (VCM)

**Fig 3:**
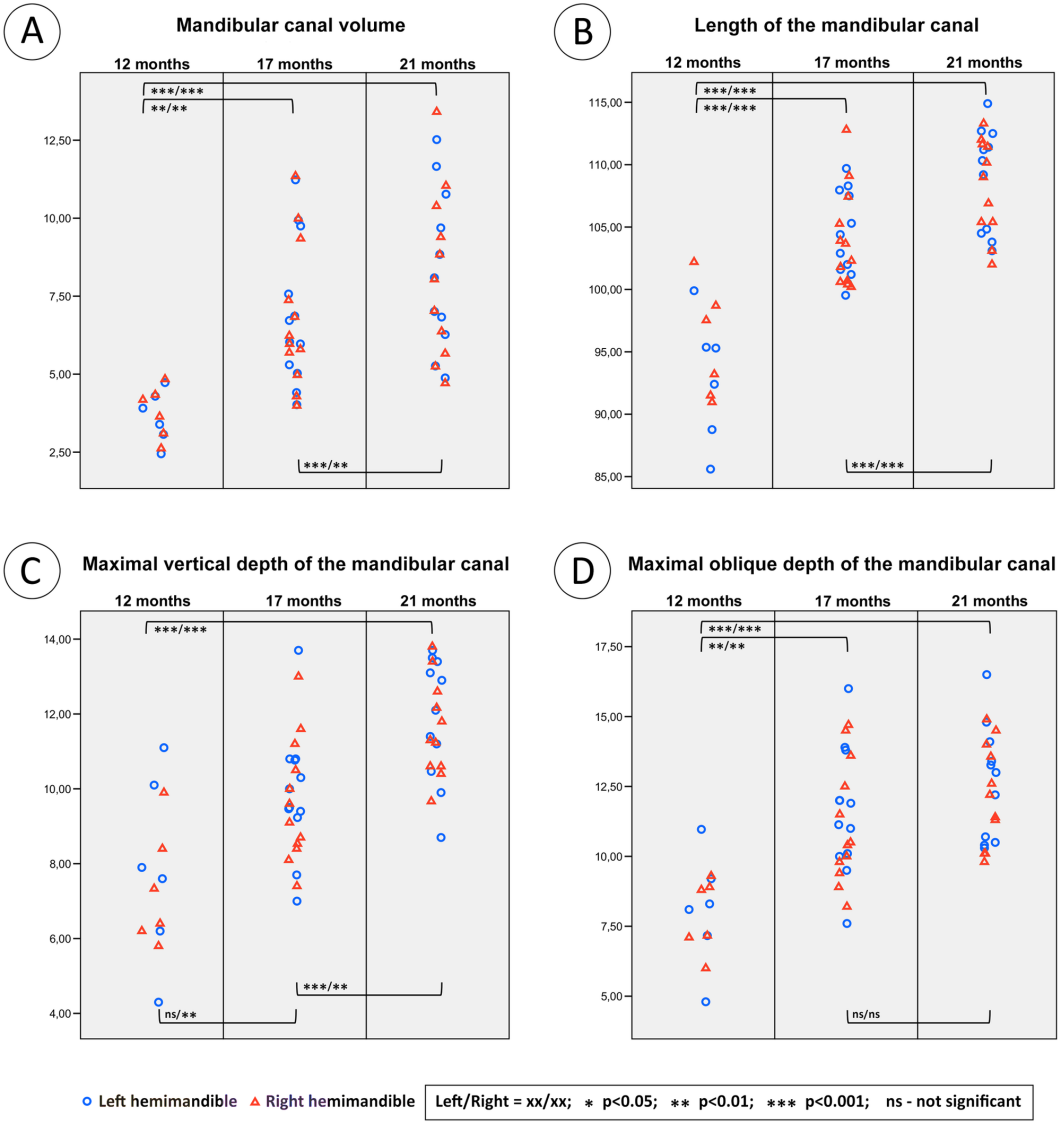
Dot plots of the measured parameters VCM (A), LCM (B), MVD (C) and MOD (D). The blue circles are for the left and the red squares for the right hemimandibles. VCM is given in millilitres, all others in millimetres.

**Fig 4:**
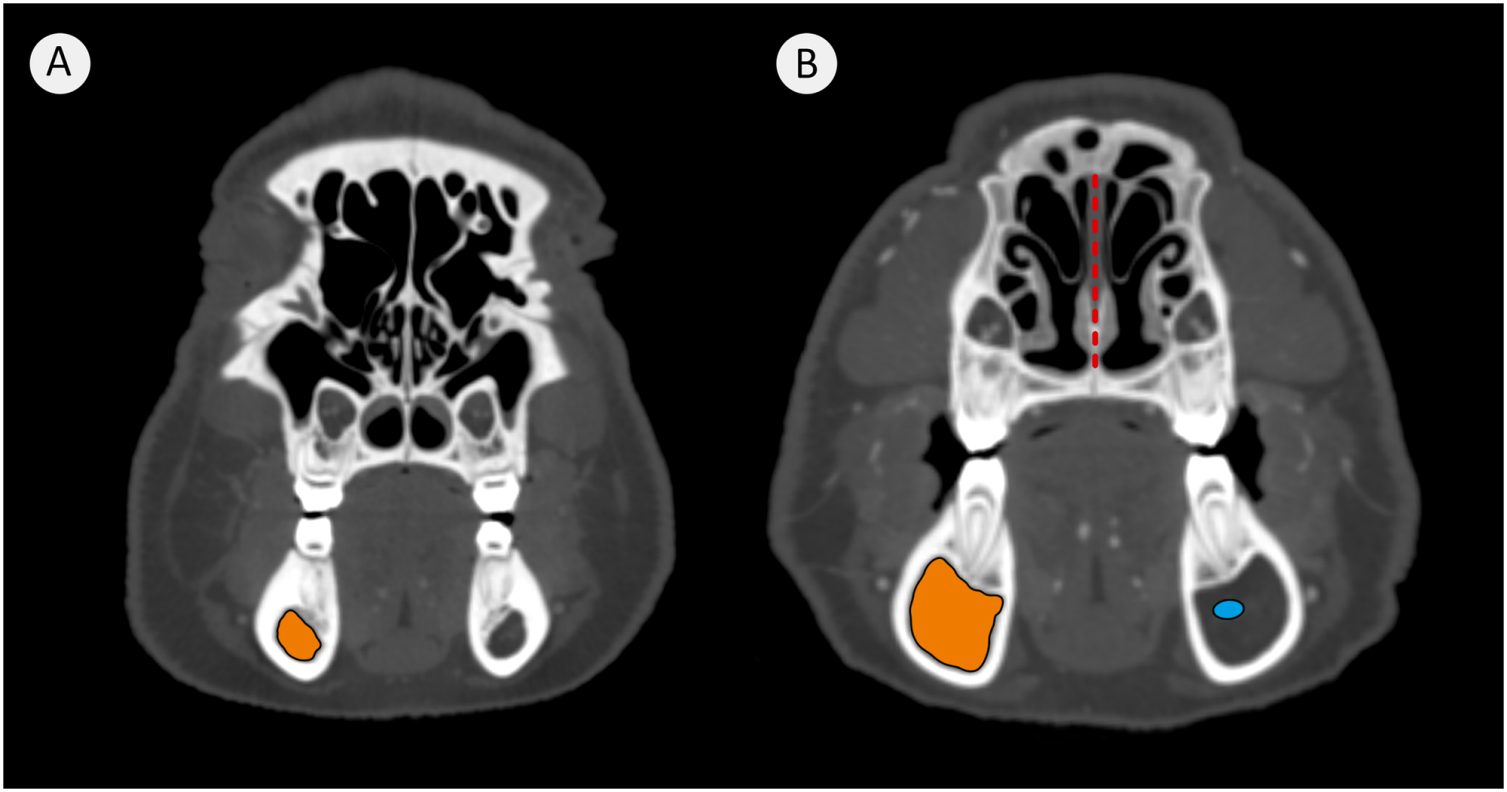
Transverse plane view at the level of the first molar tooth (M1) of two different minipigs. Where (A) is a minipig with a VCM of 4.88 mm (left) and 4.71 mm (right); (B) is a minipig with VCM of 12.52 mm (left) and 13.41 mm (right). The broken red line in (B) is the nasal septum, the orange areas are the lumina of the mandibular canals and the blue ellipse indicates the position of the inferior alveolar artery that lies beneath the inferior alveolar nerve.

The volume of both mandibular canals increases with age. All age groups differ significantly from each other (Fig 3A). The values range from 2.5 ml in 12m animals to 13.4 ml in 21m animals. Left and right canal volumes do not differ significantly from each other. Between 17 and 21 months of age, the mean increase of the canal volume was 1.2 ml for the left and 1.1 ml for the right hemimandible. Highly significant differences within the same age groups could be observed (Fig 4). VCM correlates with age but not with body mass (Table 2).

### Length of mandibular canal (LCM)

Mandibular canal length increased significantly with age (Fig 3B). Length ranges from 85.6 mm in 12m animals to 114.9 mm in 21m animals. The length of left and right canals do not differ significantly. LCM correlates with age but not with body weight (Table 2).

### Maximal vertical depth of mandibular canal (MVD)

The left canal depths of 12m and 17m animals do not differ significantly from each other. All other group comparisons were significant (Fig 3C). The values range from 4.3 mm at 12m to 13.8 mm at 21m. Left and right canal depths were similar. MVD correlates with age but not with body weight (Table 2).

### Maximal oblique depth of mandibular canal (MOD)

The maximal oblique depth of the mandibular canal increases until the age of 17 months but then does not change significantly (Fig 3D). The lowest oblique depth was found at 12m at 4.8 mm, the highest at 16.5 mm in 21m animals. Left and right oblique canal depths do not differ significantly. MOD correlates with age but not with body weight (Table 2).

### Width of mandibular canal (WCM)

**Fig 5:**
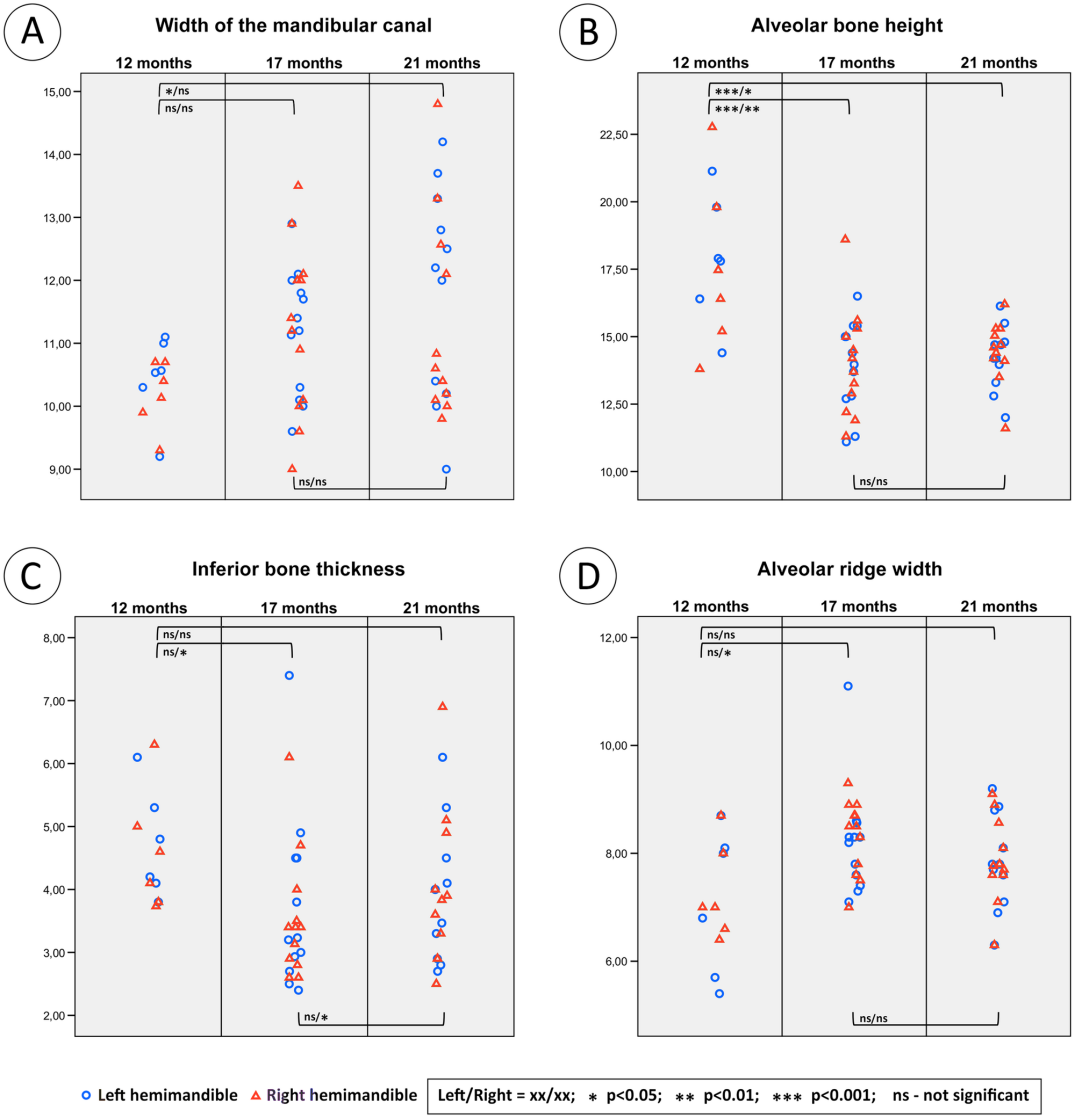
Dot plots of the measured parameters WCM (A), ABH (B), IBT (C) and ARW (D). The blue circles are for the left hemimandible and the red squares for the right. All parameters are given in millimetres.

The mean width of the mandibular canal does not change over time (Fig 5A). The only significant difference was between the left hemimandibles of 12m and 21m animals. The canal width ranged between 9.0 mm at 17m and 14.8 mm at 21m. The left and right canal widths do not differ significantly. WCM does not correlate with age or body weight (Table 2).

### Alveolar bone height (ABH)

**Fig 6:**
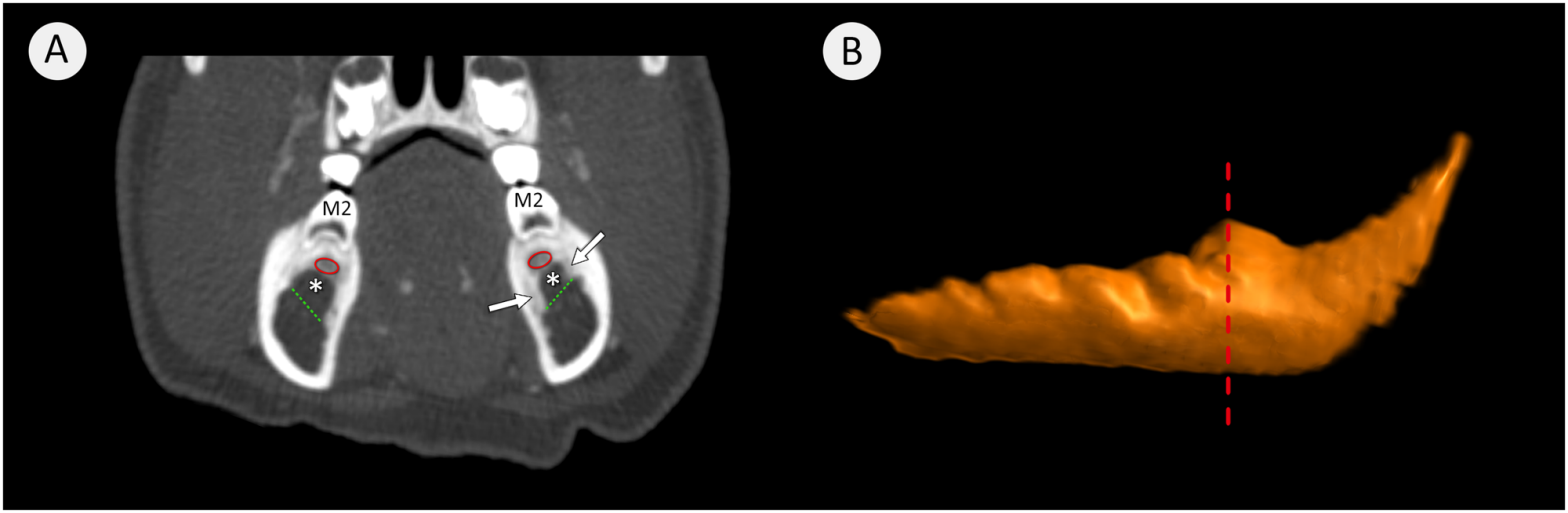
3D transverse section view and mandibular canal reconstruction. Where (A) is a transverse image at M2. The loss of spongy bone within the space around the tooth roots white arrows is shown by white stars. The residual spongy bone is demonstrated by a red oval. The green dashed lined indicate the normal superior extent of the mandibular canal. Image (B) shows the segmented mandibular canal where the dashed red line indicates the level of the second molar where image (A) was taken.

Over the course of this study, ongoing anatomical changes in the vicinity of the tooth roots were observed. The spongy bone, normally located between and beneath the tooth roots, slowly resorbs. This was particularly obvious in the molar region (Fig 6A). After this gradual loss, the residual spongy bone could be seen as a lighter grey region (red oval) directly adjacent to the tooth necks. As a result, the inferior alveolar neurovascular bundle may come into close proximity with the tooth roots.

ABH decreases between 12 and 17 months. After 17m, the height does not change significantly (Fig 5B). The distance ranges from 11.1 mm in 17m to 22.8 mm in 12m animals. There was no significant difference between left and right hemimandibles. ABH correlates negatively with age but does not correlate with body weight (Table 2).

### Inferior bone thickness (IBT)

The IBT of the left hemimandible does not differ significantly between the age groups. However, the right hemimandible has significant differences between 12m and 17m and between 17m and 21m respectively (Fig 5C). Variability can be great e.g., in 17m animals the range was 2.4 mm to 7.4 mm. Left and right hemimandibles do not differ significantly. IBT does not correlate with age or body weight (Table 2).

### Alveolar ridge width (ARW)

The alveolar ridge width does not change over time (Fig 5D). The only significant difference was seen when comparing the right hemimandibles of the 12m and 17m animals. The alveolar ridge width ranged between 5.4 mm in 12m and 11.1 mm in 17m animals. The left and right alveolar process widths do not differ significantly from each other. ARW does not correlate with age or body weight (Table 2).

### Shapes of segmented mandibular canals

**Fig 7:**
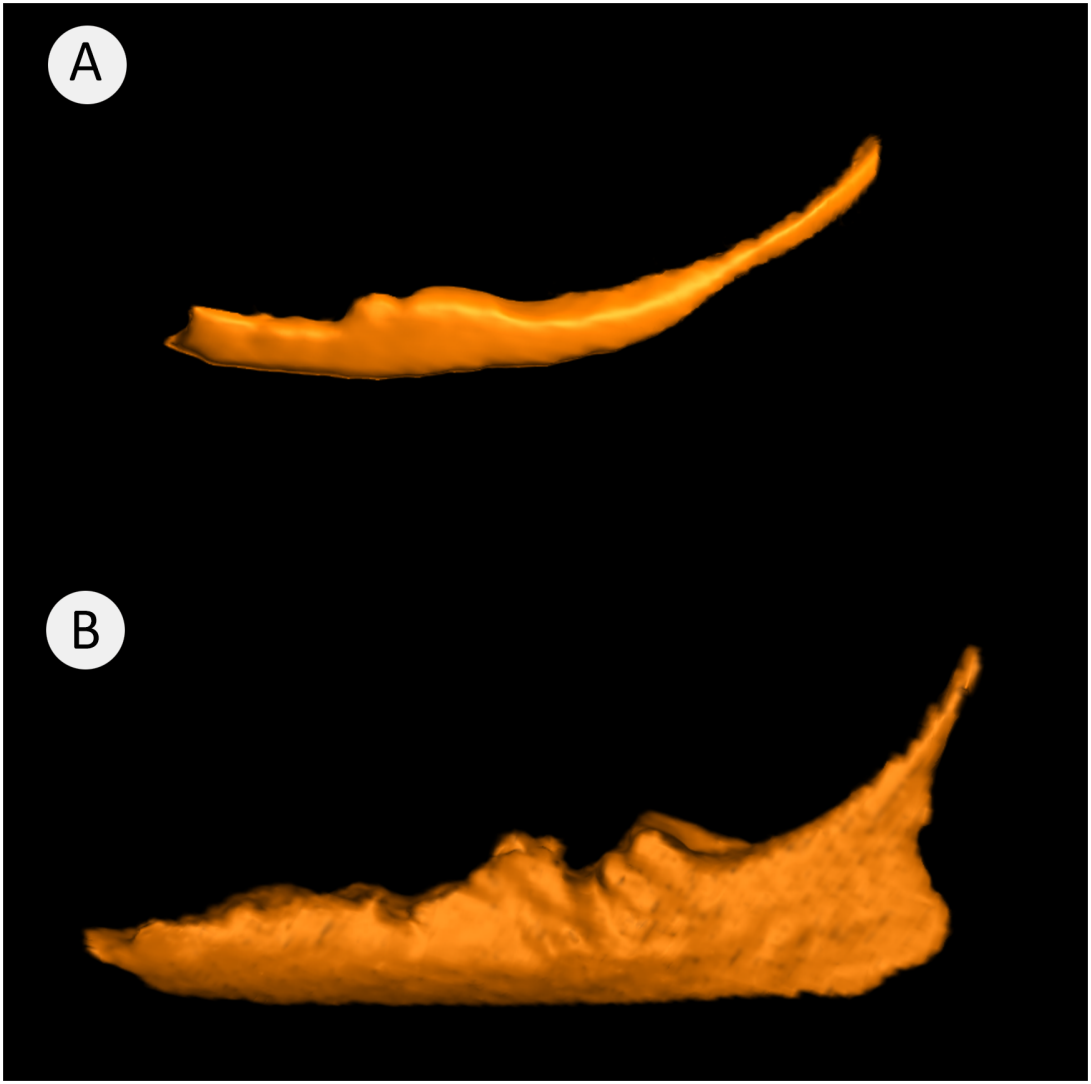
A visual comparison of mandibular canals from animals of two different age groups. Where (A) is of a 12m old minipig with a canal volume of 2.4 ml and (B) is of a 17m old animal with a canal volume of 9.9 ml. The images are scaled to size.

Segmentation composite (Fig 7A) shows a sickle-shaped mandibular canal, typical for animals of 12m of age. Segmentation composite (Fig 7B) is typical for animals of 17 and 21 months of age. It shows an obvious increase of canal depth along the whole length of the canal. The posterior border of the canal rises sharply at the level of the mandibular foramen.

**Fig 8:**
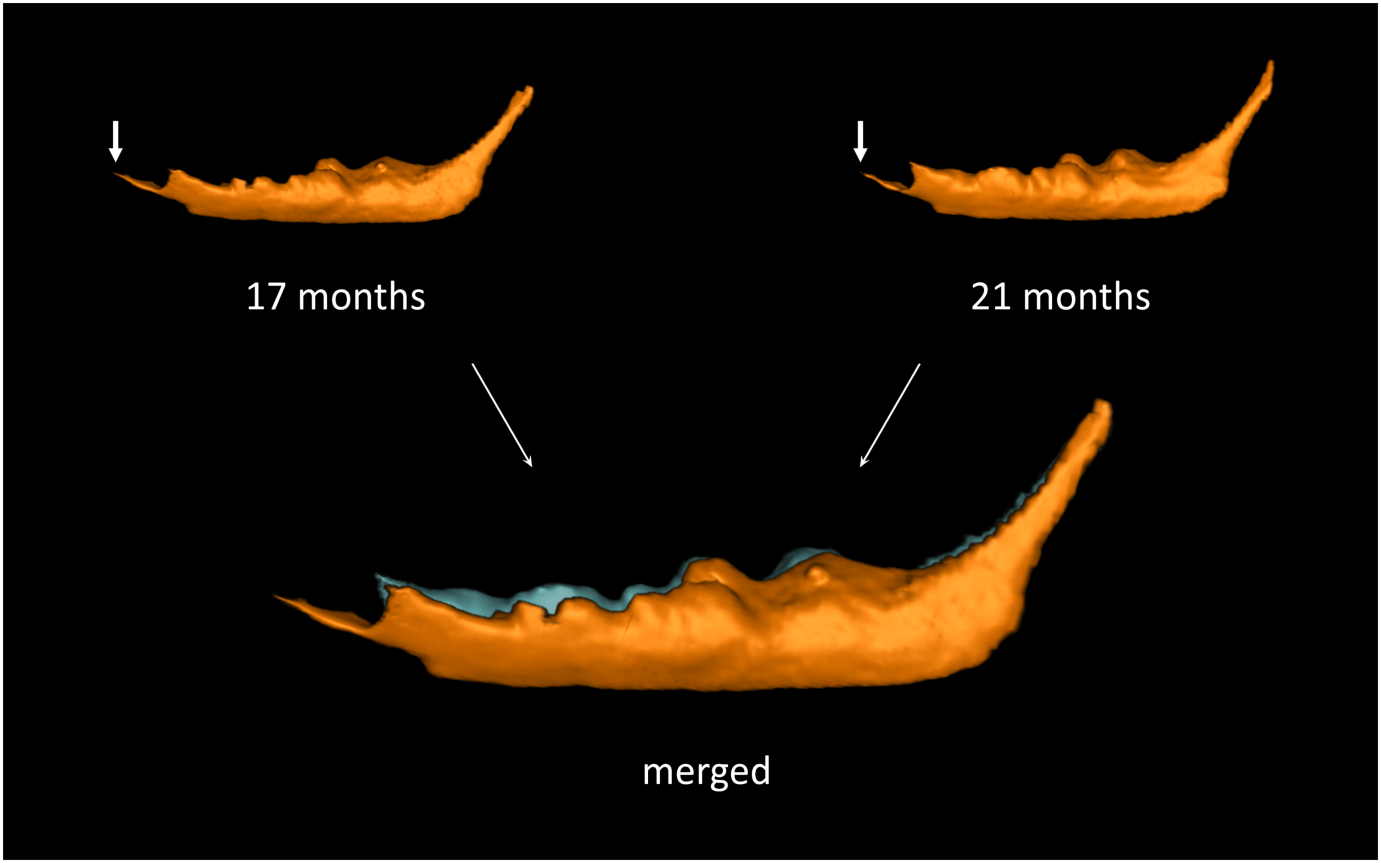
Visualization of the changes in mandibular canal volume over time. The white arrow shows the incisive canal, which is the anterior prolongation of the mandibular canal. The lower image shows the merged segmentations to enable a better visual comparison. The orange segmentation is at 17m and the bright blue at 21m.

The mandibular canal volume (Fig 8) of the same canal in the same pig, measured at 17 and 21 months of age differs. The merged volumes clearly show that the increase in volume between 17 and 21 months of age was caused mainly by a vertical superior extension in the premolar and molar regions. The ascending posterior portion of the canal near the mandibular foramen does not change in size and shape.

### Inferior alveolar neurovascular bundle

**Fig 9:**
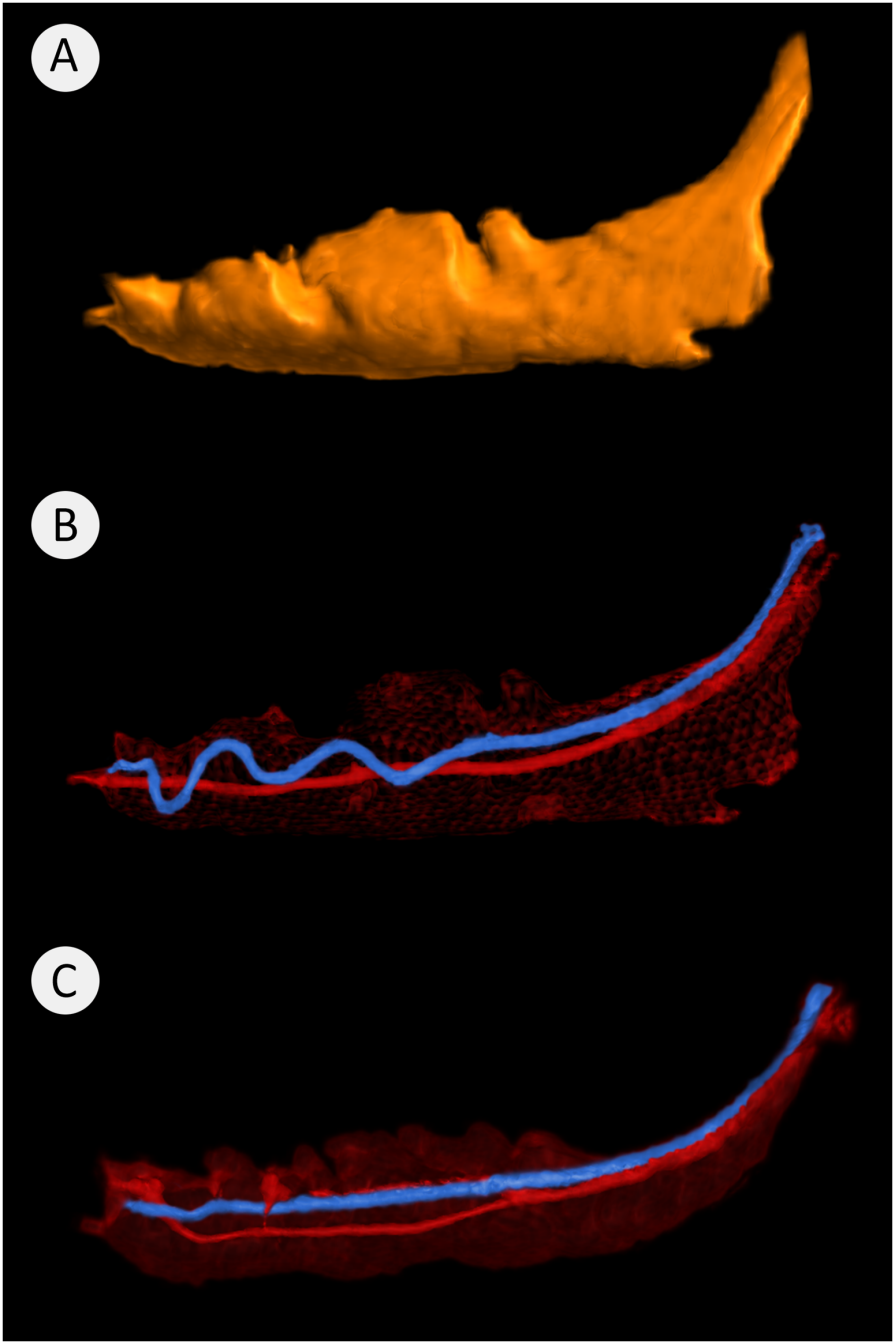
Mandibular canals and inferior alveolar vessels. Where (A) shows the typical mandibular canal outline (12.5 ml) of a 21-month-old animal. Image (B) shows the same canal but with the inferior alveolar artery (red) having a straight route and the inferior alveolar vein (blue) having an undulating route. In Image C, the inferior alveolar vessels anteriorly have a straight course.

The CT segmentations show that inferior alveolar vessels lie in the superior aspect of the mandibular canals. They may have either an undulating or a straight course (Fig 9B and 9C).

### Dissection

Both the transverse serial interrupted sections and the longitudinal excavation of the hemimandibles revealed that the inferior alveolar nerve ran in close association with the inferior alveolar blood vessels within the mandibular canal. The inferior alveolar neurovascular bundle lay adjacent the superior border of the mandibular canal in close proximity to the tooth roots. Between the mandibular foramen and the second molar, the inferior neurovascular bundle had a pronounced sheath of connective tissue. Posteriorly the neurovascular bundle lay superiorly in the midline of the mandibular canal however as it ran anteriorly it became more and more lateral (buccal).

## Discussion

This CT study of the mandibular canal of live Göttingen Minipigs aged between 12 months and 21 months found that the volume, length and depth of the canal increased over time. The width remained constant. However, as one may expect the superior region of the canal showed clear dynamic changes over the experimental period. This was particularly evident with the slow increase in volume of the mandibular canal notably associated with a gradual loss of deeper spongy bone adjacent the distal tooth roots (Fig 5). This resulted in the inferior alveolar neurovascular bundle coming into close proximity with these tooth roots. All measures of the mandibular canal within the left hemimandible were statistically similar to those of the right hemimandible. Likewise, body mass did not have an influence on any of the eight parameters reported here.

To date, the only previous study of the dimensions of the minipigs’ mandibular canal were reported by Koppe, et al in 1994 and 1997. Their study sampled 103 dried left hemimandibles of female MINI-LEWE miniature pigs. These were of 11 groups ranging from 3 days to 24 months. They found, that in contrast to the uniform diameter of the human mandibular canal that that of the minipig was enlarged in the molar and premolar regions [30, 31]. Because the MINI-LEWE pigs are considerably larger than Göttingen Minipigs their morphological dimension cannot be compared readily with each other [32].

The present study found that the volume of the mandibular canal increased between 12 and 21 months of age with a mean value of 6.7 ± 2.7 ml. There were large individual differences within each age group, for example within the 21 months old group the animal with the lowest canal volume of 4.7 ml was 14 kg heavier than the animal that had a significantly higher volume of 13.4 ml, a variation of 285% (Fig 4). These differences are not due to the length of the canal because they were identical.

As one would expect, in minipigs the length of the mandibular canal increased at a steady rate with age. The values range between 85.6 mm and 114.9 mm with a mean value of 103.8 ± 6.6 mm. Liu et al. (2009) in a study of 200 two dimensional panoramic radiographs of male and female human patients with an average age of 31.6 years reported a length of 62.46 ± 4.32 mm [33].

In this study we found that the minipigs had a mean vertical mandibular canal depth of 10.1 ± 2.2 mm with a range of 4.3 mm to 13.8 mm. Lindh et al. (1995) in a study of 71 radiographs of humans with a mean age of 76 years reported a value of 3.0 ± 0.7 mm [34]. The elderly age of the corpses in the study suggests that there may have been some mandibular atrophy present. Another study of adult Japanese cadavers reported the depth to be around 5 mm [35]. Dogs, which are also a potential animal model for dental implant testing, have a higher mean canal depth of 6.1 ± 2.1 mm than humans [36].

In the present study, all of the minipigs had an oval-shaped canal except for one individual that had a circular-shaped canal, where the mean vertical depth equals the oblique depth of the canal.

The width of the mandibular canal in the minipigs ranged from 9.0 to 14.8 mm and had a mean value of 11.2 ± 1.4 mm. However, the t-Tests show that the canal width in the minipigs does not change significantly with the age of the pigs. Humans have much narrower mandibular canal widths. Rajchel et al. (1985) reported a width of 2.0 to 2.4 mm proximal to the third molar in 45 Asian adults [37]. Ikeda et al. (1996) reported from a cadaver study a width of approximately 3.4 ± 0.5 mm midway along the length of the mandibular canal and 4.1 mm near the mandibular foramen [38]. Similar values are provided by Miller et al. (1990) in their study on 22 human mandibles. Here the mean horizontal diameter was 2.2 ± 0.5 mm with a range from 1.1 to 3.6 mm [39].

The alveolar bone height is important in the assessment of the available space for dental implant placement [40]. In the 12-month-old minipigs, it had a mean value of 17.7 ± 2.8 mm, whilst the 17 and 21 months old animals had a mean value of 13.9 ± 1.8 mm and 14.3 ± 1.2 mm respectively. Levine et al. (2007), in a morphological study of 50 adult human patients with a mean age of 42.1 years, reported a mean height of 17.4 ± 3.0 mm and a range from 8.7–23.0 mm. However, some of their subjects were partially edentulous [41]. This could result in an underestimation of the alveolar bone height due to the commencement of, or presence of, alveolar ridge atrophy [42, 43]. Liu et al. (2009) reported similar mean values with the largest height of 17.8 ± 2.2 mm [33]. Likewise, another study reported a height of 17.4 ± 3.1 mm using CT imaging on 79 Japanese patients [44]. These comparative studies show that older minipigs have a shorter alveolar bone height compared to humans. This should be taken into account when positioning a dental implant without risking the penetration of the mandibular canal [45–47], especially in the older minipigs. Dogs have an even lower mean alveolar bone height of 8.5 ± 2.6 mm [36].

In the present study of Göttingen Minipigs the mean inferior bone thickness was 4.0 ± 1.2 mm and had a range between 2.4 to 7.4 mm. Watanabe et al. reported that humans have a mean bone thickness of 9.4 ± 2.0 mm in the molar region and 15.0 ± 1.5 mm at the level of the mental foramen [44]. Another study reported a mean inferior bone thickness of 12.6 ± 1.7 mm in humans [48].

The mean alveolar ridge width of the minipigs was 7.9 ± 1.0 mm ranging between 5.4 mm in 12m and 11.1 mm in 17m animals. A classification of the alveolar ridge width published by Tolstunov (2014) states that the physiologic width in humans should be above 10 mm. Alveolar ridge widths between 6 and 8 mm are considered to indicate a mild alveolar ridge deficiency represented by a loss of buccal cortical bone and therefore an indication for alveolar distraction osteogenesis surgery [49]. When applying Tolstunov’s classification to Göttingen Minipig, their narrower alveolar ridge compared with that of healthy humans, suggests that they may be unsuitable for some experimental procedures such as dental implantation.

The morphology of the mandibular canal presented in this study aimed to visually evaluate its position, course and shape (Fig 7). In humans, the high variability in the course and shape of the mandibular canal has been described elegantly by Liu, et al. (2009), who have classified these into four distinct patterns [33]. Young Göttingen Minipigs (12m) have a rather simple, slightly curved mandibular canal (Fig 7) that closely resembles that described by Liu et al (2009) as being the most common found in humans. However, in older animals the canal becomes larger and much more complex than any seen in humans (Fig 9A). Consequently, at this stage, a similar classification is not possible for Göttingen Minipigs but is a potential subject of further studies. In the older groups over the period 17m to 21m the horizontal part of the mandibular canal i.e., within the mandibular body, enlarges vertically and has a highly convoluted superior surface. These dynamic changes are clearly seen in the merged image (Fig 8).

**Fig 10:**
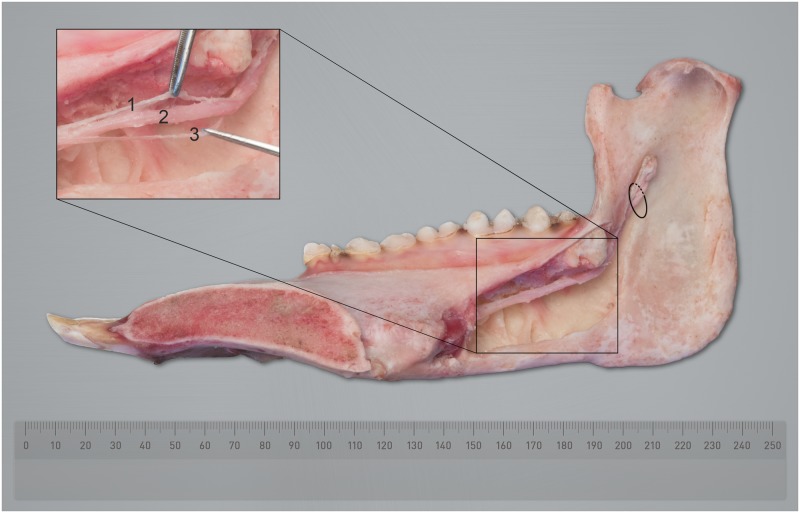
Medial longitudinal excavation of the mandibular canal of a Göttingen minipigs’ right hemimandible showing the inferior alveolar neurovascular bundle. The inset shows the inferior alveolar nerve (2) and the inferior alveolar vein (1) and artery (3). The mandibular foramen is indicated by a black ellipse.

Over most of its course, the inferior alveolar neurovascular bundle lies superiorly in the canal adjacent the distal part of the tooth roots. When one images the inferior alveolar vein, two basic patterns occur as it traverse the canal. One being a straight traverse, the other being a variable undulating route where it rises and falls as it traverses the length of the canal (Fig 9). In contrast, the inferior alveolar artery lies below the inferior alveolar vein and has a simple straight route. Dissection of the hemimandibles confirmed that the inferior alveolar nerve runs in close proximity with the blood vessels (Fig 10). Because the inferior alveolar vein lies above the inferior alveolar nerve, it is quite probable that in surgical procedures such as the implantation of dental prostheses, that damage to the vein and the nerve can occur steadily. This conclusion is supported by Pogrel et al. who described the position of the vein in humans to be in a 12 o’clock position superior to the nerve [18].

## Conclusions

This study showed that the volume of the mandibular canal in Göttingen Minipigs increases with age. This increase appears to be caused primarily by loss of deep spongy bone of the canal roof notably in the posterior premolar and in the molar regions. This results in the closer approximation of inferior alveolar neurovascular bundle to the distal tooth roots. In practice, this reduces the available space for dental implantation and could negatively affect implant stability or jeopardize the integrity of the inferior alveolar neurovascular bundle. Contrary to the increase in volume as well as its’ length and depth, the width of the mandibular canal does not change significantly over time. Despite the uniform canal width, other parameters clearly indicate progressive ongoing anatomical changes. This strongly suggests, on ethical grounds, not using minipigs younger than 21 months in experimental implant dentistry. However, paradoxically the measurements of the 12 months old pig indicate a closer alignment of the anatomy of their mandibular canal to that of humans. As such, these younger animals may be better models for implant studies.

This study also showed that the body mass of the minipigs does not have an influence on the dimensions of the mandibular canal. Consequently, choosing animals for implant surgery based on physical appearance and body dimensions is inappropriate. This, linked in with the high variability in mandibular canal dimensions in similar age cohorts, indicates that the use of CT imaging and other diagnostic imaging techniques is essential for the best selection of animals for experimental surgery as well as for probable higher success rates and thus the use of fewer animals in the long run.

## Supporting information

S1 Dataset Data of the morphometric studies of the mandibula in Göttingen Minipigs. (SAV)
